# Prefrontal cortical connectivity and coupling of infraslow oscillation in the resting human brain: a 2-channel broadband NIRS study

**DOI:** 10.1093/texcom/tgac033

**Published:** 2022-08-04

**Authors:** Sadra Shahdadian, Xinlong Wang, Shu Kang, Caroline Carter, Akhil Chaudhari, Hanli Liu

**Affiliations:** Department of Bioengineering, The University of Texas at Arlington, 500 UTA Blvd, Arlington, TX 76019, United States; Department of Bioengineering, The University of Texas at Arlington, 500 UTA Blvd, Arlington, TX 76019, United States; Department of Bioengineering, The University of Texas at Arlington, 500 UTA Blvd, Arlington, TX 76019, United States; Department of Bioengineering, The University of Texas at Arlington, 500 UTA Blvd, Arlington, TX 76019, United States; Department of Bioengineering, The University of Texas at Arlington, 500 UTA Blvd, Arlington, TX 76019, United States; Department of Bioengineering, The University of Texas at Arlington, 500 UTA Blvd, Arlington, TX 76019, United States

**Keywords:** broadband near-infrared spectroscopy, infraslow oscillation, metabolic and hemodynamic coupling, prefrontal cortical connectivity, resting state functional connectivity

## Abstract

The resting-state infraslow oscillation (ISO) of the cerebral cortex reflects the neurophysiological state of the human brain. ISO results from distinct vasomotion with endogenic (E), neurogenic (N), and myogenic (M) frequency bands. Quantification of prefrontal ISO in cortical hemodynamics and metabolism in the resting human brain may facilitate the identification of objective features that are characteristic of certain brain disorders. The goal of this study was to explore and quantify the prefrontal ISO of the cortical concentration changes of oxygenated hemoglobin (Δ[HbO]) and redox-state cytochrome *c* oxidase (Δ[CCO]) as hemodynamic and metabolic activity metrics in all 3 E/N/M bands. Two-channel broadband near-infrared spectroscopy (2-bbNIRS) enabled measurements of the forehead of 26 healthy young participants in a resting state once a week for 5 weeks. After quantifying the ISO spectral amplitude (SA) and coherence at each E/N/M band, several key and statistically reliable metrics were obtained as features: (i) SA of Δ[HbO] at all E/N/M bands, (ii) SA of Δ[CCO] in the M band, (iii) bilateral connectivity of hemodynamics and metabolism across the E and N bands, and (iv) unilateral hemodynamic–metabolic coupling in each of the E and M bands. These features have promising potential to be developed as objective biomarkers for clinical applications in the future.

## Introduction

### Infraslow oscillation of the human brain

The human brain plays a major role in oxygen and glucose consumption despite its relatively low weight compared to other organs (Raichle and Gusnard 2002; Magistretti and Allaman 2013). The high levels of consumption are due to the high metabolic activity of neurons, which is modulated by the oxygenated blood supply and cerebral metabolism (Zhu et al. 2009; Mateo et al. 2017). Many studies have focused on investigating the mechanism of cerebral metabolic activity and have found vasomotion to be a major source of metabolic and hemodynamic modulations (Brown et al. 2002; Klocke 2010; Rivadulla et al. 2011; Vermeij et al. 2014; van Helden and Imtiaz 2019). Vasomotion is a spontaneous oscillation that originates from the blood vessel wall with an infraslow oscillation (ISO) of 0.005–0.2 Hz (Wiedeman 1957; Buerk and Riva 1998). In addition, a correlation is found between the ISO of cerebral metabolic activities and human cognitive functions (Bosch et al. 2017). Furthermore, vasomotion malfunction has been observed in older adults and in patients with different diseases, such as atherosclerosis (Vita and Keaney Jr 2002), cardiovascular disease (Deanfield et al. 2007), and Alzheimer’s disease (Marco et al. 2015). Thus, it may be beneficial to quantify and characterize cerebral metabolism in the ISO range, which may provide better insight into neurophysiological mechanisms and discover features that differ between healthy humans and patients with brain disorders.

Relaxation–contraction cycles of blood vessel walls have been shown to be the driving force for the infraslow rhythms of cerebral hemodynamic oscillations, independent of respiration and heartbeat (Bracic and Stefanovska 1998; Stefanovska et al. 1999; Bernjak et al. 2008; Vermeij et al. 2014). Three intrinsic frequency components of infraslow cerebral hemodynamic rhythms have been found to correspond to the specific physiological and biochemical activities of the vascular wall layers (Mustari et al. 2018). These frequency bands consist of (i) endogenic (0.005–0.02 Hz), (ii) neurogenic (0.02–0.04 Hz), and (iii) myogenic (0.04–0.2 Hz) (Kvernmo et al. 1999; Zhang et al. 2002; Newman et al. 2009) rhythms. The endogenic band corresponds to dilation–contraction cycles in the endothelial layer affected by the release of potent vasoactive factors, such as nitric oxide (NO), free radicals, prostacyclin, endothelium-derived hyperpolarizing factor, and endothelin (Faraci and Heistad 1998; Girouard and Iadecola 2006). Oscillation in releasing vasoactive ions and neurotransmitters from neurons leads to modulation of the vessel dilation–contraction cycles in the neurogenic band (Schmidt et al. 1993). Rhythmic myogenic activity, on the other hand, occurs as a result of the relaxation and contraction of vascular wall smooth muscle cells (Newman et al. 2009). Such hemodynamic ISO can be detected by different measurement modalities, such as functional magnetic resonance imaging (fMRI) (Damoiseaux et al. 2006), transcranial cerebral Doppler (TCD) (Lang et al. 1999), and functional near-infrared spectroscopy (fNIRS) (Bosch et al. 2017). However, these methods are not capable of concurrently monitoring the metabolic rhythms originating in the mitochondria. As mitochondria play a major role in cerebral metabolism and vasomotion, detecting mitochondrial activity and ISO is essentially important (Tang et al. 2014).

### Exploration of the prefrontal cortical connectivity and coupling of ISO

The bilateral prefrontal connectivity of the human brain with respect to certain neurophysiological functions reflects the level at which the lateral sides of the prefrontal cortex oscillate synchronously or coherently. Therefore, a higher level of connectivity represents a bilaterally or globally driven oscillation, while a lower level of connectivity denotes locally driven activity (Obrig et al. 2000). On the other hand, unilateral hemodynamic–metabolic coupling indicates how the supply–demand relationship between local oxygenated hemodynamics and metabolism is regulated. Any impaired, abnormal, or diminished bilateral connectivity and/or unilateral/local coupling of the prefrontal ISO should reflect or result from neurological diseases or brain disorders. This is because prefrontal cortex activity is closely associated with human cognition; many studies have provided evidence (Motzkin et al. 2011; Racz et al. 2017; Sampath et al. 2017; Yu et al. 2020; Jobson et al. 2021). Thus, it is desirable to quantify prefrontal cortical connectivity and coupling of ISO in the human brain, which may be closely associated with normal or abnormal brain states and may be developed for clinical applications in the near future.

### Broadband near-infrared spectroscopy and resting-state analyses

Broadband NIRS (bbNIRS) has been investigated for more than 2 decades (Matcher et al. 1995; Gagnon et al. 1996; Pogue and Patterson 1996; Uludağ et al. 2004; Kolyva et al. 2012) and accepted as a reliable tool to quantify changes of oxygenated and deoxygenated hemoglobin concentrations (∆[HbO] and ∆[HHb], respectively) as well as redox-state cytochrome *c* oxidase (CCO) concentration (∆[CCO]) based on absorption and scattering of NIR light by these chromophores (Kolyva et al. 2012; Bainbridge et al. 2014; Bale et al. 2014). In particular, cytochrome *c* oxidase is the terminal enzyme in the mitochondrial respiratory chain that catalyzes the reduction of oxygen for energy metabolism (Eells et al. 2004; Wong-Riley et al. 2005; Gonzalez-Lima et al. 2014; Gonzalez-Lima and Auchter 2015). Because redox CCO has a light absorption peak at ~800 nm, bbNIRS can quantify Δ[CCO] and enable us to reveal the metabolic state of a tissue (Kolyva et al. 2012; Bainbridge et al. 2014; Bale et al. 2014). However, since the concentration of CCO is much smaller than those of oxy- and deoxy-hemoglobin in living tissues, accurate estimation of ∆[CCO] requires a multispectral approach (Tachtsidis et al. 2010; Bainbridge et al. 2014; Bale et al. 2014). In the past several years, our group has successfully quantified redox ∆[CCO] in response to photobiomodulation using 1-channel or 2-channel bbNIRS (2-bbNIRS) taken on the human forearm or forehead (Wang et al. 2016; Wang et al. 2017; Pruitt et al. 2020; Pruitt et al. 2022).

However, most studies in the field of either fNIRS or bb-NIRS are based on time domain analyses and are often performed under task-evoked brain states (Boas et al. 2014; Tian et al. 2014; Yennu et al. 2016). Numerous articles on fNIRS-derived resting-state connectivity have been based only on hemodynamic (∆HbO) oscillations (Niu and He 2014; Cai et al. 2018; Hu et al. 2021). Little or no report could be found on the frequency domain analysis of bb-NIRS measurements in the resting human brain. It is also unknown whether 2-bbNIRS can facilitate the characterization of prefrontal connectivity and coupling in the brain.

In the present exploratory study (Hallingberg et al. 2018), we hypothesized that 2-bbNIRS, along with frequency domain analysis, enables us to quantify prefrontal cortical connectivity and coupling of ISO in the resting human brain. Specifically, the features analyzed from the 2-bbNIRS time series included (i) resting-state spectral amplitude (SA) of bilateral cortical hemodynamic and metabolic (i.e. SA_HbO__*i* and SA_CCO__*i*) activities, where *i* represents either the left or right prefrontal region; (ii) bilateral hemodynamic connectivity (bCON_HbO_); (iii) bilateral metabolic connectivity (bCON_CCO_); and (iv) coupling between cerebral hemodynamic and metabolic activities on the unilateral side (uCOP_HbO-CCO__*i*) of the prefrontal cortex over the 3 ISO frequency bands. By the end of this exploratory study, we would support this hypothesis by presenting relatively stable and consistent values for these features in healthy young humans, revealing the translation potential of these features for future clinical applications.

## Materials and methods

### Participants

Thirty-one healthy human subjects were recruited from the local community at the University of Texas, Arlington. They were screened using the same inclusion criteria as those used in the previous studies (Wang et al. 2017; Pruitt et al. 2020). Each participant had 5 visits, separated by at least 7 days. Because of the high sensitivity of bbNIRS to motion artifacts, 5 subjects with excessive motion during one or more of the 5 experiments were excluded from the data. After exclusion, a total of 26 young and healthy humans (14 males and 12 females, mean ± SD age = 22.4 ± 2.3 years) participated in the 5-visit experiments. The study protocol complied with all applicable federal guidelines and was approved by the Institutional Review Board (IRB) of the University of Texas at Arlington. Informed consent was obtained from all participants.

### Experiment setup and protocol

**Fig. 1:**
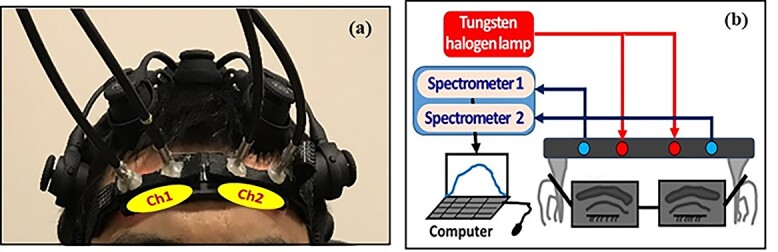
a) Dual-mode (bbNIRS and EEG) head probe setup, showing 2 separate channels with 2 sets of fiber bundles that were connected to (b) the 2-channel bbNIRS. While an EEG cap on the head is observable, the EEG data are not the topic/subject of this paper. The bbNIRS datasets used for this study were taken during 7-min eyes-closed conditions with the setup shown.

The data analyzed in this study were obtained from single-mode, resting-state, bilateral measurements with 2-channel bbNIRS, which is one of the dual-mode (i.e. bbNIRS and EEG) modalities. Specifically, a 2-channel bbNIRS probe (Fig. 1a) was placed bilaterally on the forehead of the participants to acquire prefrontal ISO signals of Δ[HbO] and Δ[CCO] at rest. The 2-channel system consisted of 2 branches of a broadband white light source (Model 3900e, Illumination Technologies, NY, USA) and 2 CCD array spectrometers (QEPRO, Ocean Optics Inc., Orlando, FL, USA) as light detectors (Fig. 1b). The 2 bbNIRS recording channels were positioned symmetrically on the subject’s forehead (visual judgment). Each channel consisted of one fiber bundle for light delivery to the forehead and another for backscattered light collection from the brain tissue, with a source-detector separation of 3 cm. A 2-channel probe holder was designed and 3D printed with a flexible material to ensure comfortable and firm attachment of the fiber bundles to the forehead skin, accommodating each participant’s forehead curvature. The probe holder was fastened to each participant’s forehead with hook-and-loop fasteners, and adhesive medical tape was applied to the probe–skin interface to hold the probe on the forehead more steadily (without tightening the fastener too much), thus reducing motion artifacts.

Regarding the measurement protocol, after the consent form was signed, each participant was instructed to sit comfortably on a chair, followed by a dual-mode probe placement on the participant’s head firmly. Then, the 2-channel bbNIRS (and EEG) started to record data at a rate of 1.5 s per temporal point (i.e. 0.67 Hz) during the 7-min resting state while the participant kept their eyes closed without falling asleep.

### Data analysis

**Fig. 2:**
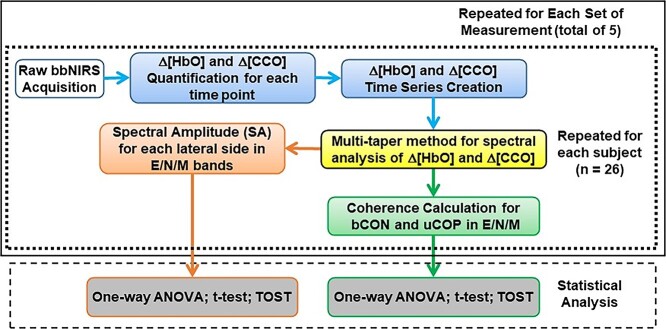
A data processing flow chart with 5 steps. Step 1: Δ[HbO] and Δ[CCO] quantification at each time point and time series (blue boxes). Step 2: Amplitude and phase decomposition using multi-taper method (yellow box). Step 3: Quantification of SAs for endogenic, neurogenic, and myogenic (E/N/M) frequency bands (orange box). Step 4: Determination of 4 types of coherences for each E/N/M bands (green box). Steps 1–4 were repeated for each of 26 participants (outlined by the dotted box) and then for 5 sets of the measurements (outlined by the solid box). The bottom dashed box marks Step 5, showing several statistical analyses, including one-way ANOVA, paired *t*-tests, and 2 one-sided tests (TOST) used to identify group-level features for SA and respective coherence parameters (gray box).

After 2-bbNIRS data acquisition, the data processing steps included both time and frequency domain analyses, as outlined in Fig. 2, in 5 steps. Step 1 (blue boxes in Fig. 2) was to obtain the Δ[HbO] and Δ[CCO] time series after converting the raw data to Δ[HbO] and Δ[CCO] at each time point. Step 2 (the yellow box in Fig. 2) involved performing frequency domain analysis using the multitaper method (MTM) that facilitated the following 2 steps to investigate the cerebral hemodynamic and metabolic ISO of the human prefrontal cortex in the resting state. Step 3 (the orange box) was to quantify SAs of Δ[HbO] and Δ[CCO] (i.e. SA_HbO__*i* and SA_CCO__*i*) in the endogenic, neurogenic, and myogenic (E/N/M) frequency bands measured on each lateral prefrontal site, where the subscript of “*i*” labels either “L” or “R” for the left or right forehead. Step 4 (green box) was used to perform coherence analysis and to determine (i) bilateral connectivity for Δ[HbO] and Δ[CCO] (i.e. bCON_HbO_ and bCON_CCO_) of the human forehead and (ii) unilateral cerebral hemodynamic–metabolic coupling (uCOP_HbO-CCO__*i*) for each lateral prefrontal cortex. Steps 1–4 were repeated for each of the 26 participants and then for 5 sets of measurements. Step 5: One-way ANOVA was performed to demonstrate no significant difference among the 5 measurements for each of the bilateral SA, bilateral connectivity, or unilateral coupling parameters (i.e. SA_HbO__*i*, SA_CCO__*i*, bCON_HbO_, bCON_CCO_, uCOP_HbO-CCO__*i*) in each of the E/N/M bands.

### Step 1: quantification of Δ[HbO] and Δ[CCO] time series

As mentioned in the Introduction section, bbNIRS has been studied for more than 2 decades (Matcher et al. 1995; Gagnon et al. 1996; Pogue and Patterson 1996; Uludağ et al. 2004; Kolyva et al. 2012) and is well accepted as a reliable tool for quantifying cortical ∆[HbO], ∆[HHb], and ∆[CCO] based on the modified Beer–Lambert law (MBL) (Kolyva et al. 2012; Bainbridge et al. 2014; Bale et al. 2014). Following the same approach, we selected the spectral range of 780–900 nm from the recorded optical spectrum at each time point and quantified prefrontal Δ[HbO] and Δ[CCO] based on MBL and multiple linear regression analysis with a low-pass filter at 0.2 Hz (Boas et al. 2014). Detailed derivations and steps can be found in Refs. (Wang et al. 2016, 2017). After repeating the concentration quantification at all recorded time points, we obtained a time series of Δ[HbO] and Δ[CCO] for the 7-min resting-state period at a sampling frequency of 0.67 Hz. The spectral range of 780–900 nm estimates the chromophore concentration with a low level of error propagated from noise (Truong et al. 2022).

### Step 2: MTM for spectral analysis of Δ[HbO] and Δ[CCO]

The MTM (Park et al. 1987; Babadi and Brown 2014) is a well-known time–frequency analysis for a time series. Specifically, multiple tapers, mainly Slepian sequences, are used to taper the recorded signal in the time domain before performing the Fourier transform to provide a frequency domain spectrum (Park et al. 1987; Babadi and Brown 2014). This method maintains a reasonably high spectral resolution while reducing spectral noise. In this study, the MTM enabled us to decompose the amplitude and phase of the Δ[HbO] and Δ[CCO] time series obtained from both bbNIRS channels. Specifically, we utilized several functions (including “ft_freqanalysis” and “ft_connectivityanalysis”) available in the FieldTrip toolbox (Oostenveld et al. 2011; Popov et al. 2018) to perform MTM operations. Section A in the Supplementary Material explains the 2 functions of “ft_freqanalysis” and “ft_connectivityanalysis” and presents a detailed flowchart (Supplementary Fig. S1) to illustrate the calculations for SA.

### Step 3: quantification of SA in E/N/M bands

One of the outputs of MTM is the power spectral density (PSD) smoothed over a given frequency range. In this study, smoothed PSDs of Δ[HbO] and Δ[CCO] over a 7-min resting period were obtained across E/N/M frequency bands. For a 7-min measurement duration, the spectral (or frequency) resolution was 1/(7 min) = 1/(7 × 60 s) = 0.0024 Hz. Accordingly, the signal spectral power at each PSD frequency was obtained by multiplying the PSD value by the spectral resolution at the respective frequency. Next, by taking the square root of the spectral power, we were able to attain a spectrum of ISO amplitude versus frequency between 0.005 and 0.2 Hz (as selected in Step 1). Finally, we obtained the mean SAs over each ISO band for Δ[HbO] and Δ[CCO]. The methodological steps are expressed as follows:

(1) \begin{equation*} \mathrm{amplitude}(f)=\sqrt{\mathrm{power}(f)}=\sqrt{\mathrm{PSD}(f)\times \Delta f} \end{equation*}

(2) \begin{equation*} \mathrm{SA_{HbO}}\_i=\mathrm{mean}\ \mathrm{amplitude}(f)\ \mathrm{of}\ \Delta \left[\mathrm{HbO}\right]\ \mathrm{over}\ \mathrm{the}\ i\mathrm{th}\ \mathrm{band}, \end{equation*}

(3) \begin{equation*} \mathrm{SA_{CCO}}\_i=\mathrm{mean}\ \mathrm{amplitude}(f)\ \mathrm{of}\ \Delta \left[\mathrm{CCO}\right]\ \mathrm{over}\ \mathrm{the}\ i\mathrm{th}\ \mathrm{band}, \end{equation*}

where PSD(*f*), power(*f*), and amplitude(*f*) are the frequency-dependent spectra of PSD, power, and amplitude, respectively; *i* represents the *i*th band for E/N/M frequencies (i.e. *i =* E, N, M) on each side of the participant’s forehead.

### Step 4: hemodynamic and metabolic connectivity and coupling by coherence

In theory, brain connectivity measures rely on the amplitude and/or phase of the signal recorded from each channel to quantify the level at which each pair of signals interact with each other. Based on the mathematical definition of the connectivity measure, the correlation between the phases and/or amplitudes of 2 time series (recorded by two respective channels) can be interpreted as the functional connectivity/coupling of these time series (Friston 1994; Bastos and Schoffelen 2015). In contrast, the counterpart of the time domain cross-correlation calculation is coherence in the frequency domain, which can be used to facilitate or quantify the cerebral connectivity in this study. The coherence coefficient is a normalized number between 0 and 1 without any unit and is expressed as a function of frequency, ω, as follows (Bastos and Schoffelen 2015):

(4) \begin{equation*} co{h}_{xy}\left(\omega \right)=\frac{\left|{S}_{xy}\left(\omega \right)\right|}{\sqrt{S_{xx}\left(\omega \right){S}_{yy}\left(\omega \right)}}, \end{equation*}

where *S_xx_* and *S_yy_* indicate the power estimates of the signals *x* and *y*, respectively, and *S_xy_* represents the averaged cross-spectral density term of the 2 signals. These terms can be calculated using the complex values obtained from the MTM method (Percival and Walden 1993; Babadi and Brown 2014).

In the next step of spectral analysis, we quantified 4 pairs of spectral coherence for the resting-state human forehead: (i) bilateral coherence of Δ[HbO] to represent bilateral hemodynamic connectivity (bCON_HbO_), (ii) bilateral coherence of Δ[CCO] to represent bilateral metabolic connectivity of (bCON_CCO_), (iii) unilateral coherence between Δ[HbO] and Δ[CCO] on the left, and (iv) right side of the forehead to designate hemodynamic–metabolic coupling on the respective prefrontal cortex (i.e. uCOP_HbO-CCO_L_ and uCOP_HbO-CCO_R_). In practice, the function of “ft_connectivityanalysis,” available in the FieldTrip toolbox (Oostenveld et al. 2011; Popov et al. 2018), was used to facilitate these coherence spectra, followed by band averaging within each of the 3 (E/N/M) frequency bands of the ISO. The flow chart (Supplementary Fig. S1) in the Supplementary Material offers graphical steps for calculating coherence.

### Step 5: statistical analyses for ISO features

The aforementioned steps were repeated for each of the 2 bbNIRS channels for all subjects during each of the 5 visits. Three stages of statistical analyses were performed for SA_HbO_ (or SA_CCO_):

(1) ANOVA was performed to prove that there was no significant difference in SA_HbO_ (or SA_CCO_) among the 5 measurements. This set of ANOVA tests was performed for each of the Δ[HbO] (or Δ[CCO]) metrics on the bilateral channels.
(2) A set of paired *t*-tests was performed to compare the bilateral values of grand-averaged SA_HbO_ (or SA_CCO_) over 5 repeated measurements from all 26 participants at all 3 E/N/M bands.
(3) The two one-sided test (TOST) analysis was utilized to evaluate the equivalence of the features that did not show a significant difference between bilateral values for SA_HbO_ (or SA_CCO_). Details of the equivalence test using TOST can be found in Section B of the Supplementary Material.

**Fig. 3:**
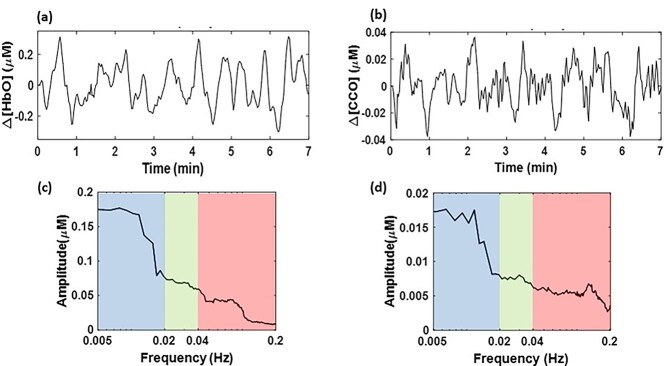
a, b) An example of time domain representation of Δ[HbO] and Δ[CCO] signals, respectively, with a frequency band of 0.005–0.2 Hz over a period of 7 min. This set of time series was derived after processing Step 1 from one channel of the subject’s dataset. c, d) The frequency domain SAs for Δ[HbO] and Δ[CCO], respectively, quantified using Steps 2 and 3. Blue, green, and red indicate endogenic, neurogenic, and myogenic bands, respectively.

After these stages of analyses, all bilaterally equivalent values of SA_HbO_ (or SA_CCO_) at each E/N/M band were reported as features for the prefrontal hemodynamic (or metabolic) SAs.

Similar statistical analyses of the 3 stages were performed on 4 coherence metrics that were band-averaged over all the subjects for each of the 5 visits. In ANOVA tests, after obtaining the bilateral connectivity indices for bCON_HbO_ (or bCON_CCO_) for all 3 E/N/M bands, a one-way ANOVA was performed to assess the similarity among the 3 bands, followed by Tukey’s post hoc test or TOST to detect statistically different or equivalent bCON_HbO_ (or bCON_CCO_) indices, respectively, across the 3 bands. Finally, grand-averaged uCOP_HbO-CCO_ indices on the 2 lateral (left and right) sides were compared using a set of paired *t*-tests for all 3 E/N/M bands. In case of no significant difference between the left and right uCOP_HbO-CCO_ in any of the 3 bands, TOST was performed to test and prove the equivalence of the bilateral values of uCOP_HbO-CCO_. Then, the averaged value of uCOP_HbO-CCO_ was reported as a feature for prefrontal, resting-state hemodynamic–metabolic coupling.

## Results

The hypothesis of this study was that bilateral hemodynamic and metabolic connectivity and unilateral coupling of the ISO in the resting human forehead can be quantified using 2-bbNIRS and may serve as measurable features reflecting the prefrontal brain state. To prove or support this, we took 7-min, resting-state, 2-bbNIRS measurements from the forehead of 26 young and healthy participants (after exclusion of 5 subjects). The analyzed results focused on (i) SAs, (ii) bilateral coherence, and (iii) unilateral coherence among 4 time series of Δ[HbO] and Δ[CCO] signals obtained from the prefrontal cortex.

### Time series of Δ[HbO] and Δ[CCO] versus their spectral analysis

After fitting the MBL with the spectral data of 2-bbNIRS (Step 1), we obtained a 7-min time series of Δ[HbO] and Δ[CCO] from each lateral side of the forehead of each participant. As an example, Fig. 3a and b shows time profiles of Δ[HbO] and Δ[CCO] derived from one channel of one subject’s dataset; their time series fluctuated around 0 between ±0.3 μM and ±0.04 μM, respectively. After performing the spectral analysis (Step 2) and quantification of SA (Step 3), we obtained SA values for both Δ[HbO] and Δ[CCO], as shown in Fig. 3c and d, respectively, where the 3 frequency bands (E/N/M) are color-shaded. In addition, Section C in the Supplementary Material shows an example of the Δ[HbO] time series from one channel of 2-bbNIRS of a subject’s dataset. This figure illustrates how different ISO waveforms in the 3 E/N/M bands contribute to the composition of the wideband (0.005–0.2 Hz) original signal.

### ISO SAs of prefrontal Δ[HbO] and Δ[CCO] in the resting brain

**Fig. 4:**
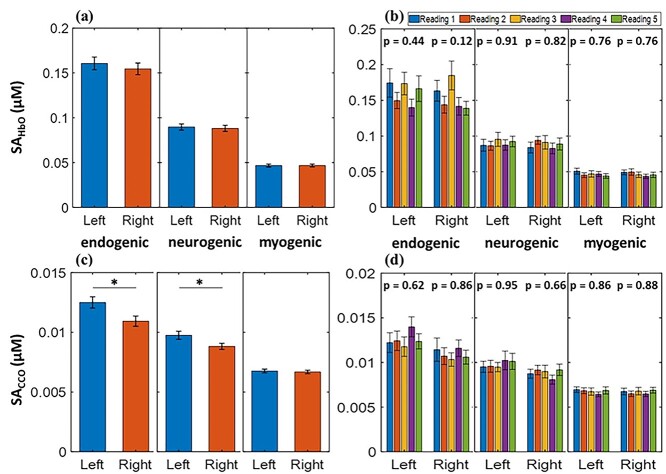
Resting-state prefrontal SA_HbO_ (in μM) of the left and right forehead averaged over (a) a combined set of grand/total measurements (*n* = 130) and (b) each individual set of 5 measurements (*n* = 26 per set) at endogenic (E; 0.005–0.02 Hz), neurogenic (N; 0.02–0.04 Hz), and myogenic (M; 0.04–0.2 Hz) frequency bands. Similarly, resting-state prefrontal SA_CCO_ (in μM) of the left and right forehead averaged over (c) the combined set of measurements (*n* = 130) and (d) each individual set of 5 measurements (*n* = 26 per set) at E/N/M bands. *P*-values shown for each group of bars in (b) and (d) represent ANOVA results. All error bars are based on the standard error of the mean. ^*^P < 0.05.

Figure 4a and c shows the SA_HbO_ and SA_CCO_ values in the E band, which are dominant over those in the other two bands. Furthermore, the paired *t*-test results demonstrated no significant difference in SA_HbO_ between the 2 prefrontal regions across all 3 E/N/M bands. These observations are in good agreement with those of a recent and independent study by our group (Wang et al. 2022), which utilized a completely different bbNIRS system and setup from a different cohort of participants. In addition, the data processing algorithms used to obtain the SA_HbO_ differed between the 2 studies. Similarly, SA_CCO_ values from both prefrontal cortices were statistically equivalent in the M band. However, the SA_CCO_ values in the left prefrontal cortex were significantly higher than those in the right prefrontal cortex in both the E- and N-bands.

To evaluate the consistency and stability of the 2-bbNIRS measurements, 5 sets of derived/quantified SA_HbO_ and SA_CCO_ values were determined and plotted in Fig. 4b and d, respectively. One-way ANOVA was performed to assess significant differences among the five measurements. The analysis outcomes showed no statistically significant differences among the 5 datasets for each of the three frequency bands.

Specifically, the second and third columns from the left of Table 1 represent the grand averages of SA_HbO_ values (as shown in Fig. 4) over all experiments (*n* = 130) taken from the left and right prefrontal cortices of the 26 participants across the 3 ISO frequency bands. The fourth and fifth columns list the *P*-values and *P*_TOST_ obtained from the paired *t*-tests and TOST analysis, respectively, between the left and right SA_HbO_ values. This table indicates that left and right SA_HbO_ values were statistically equivalent in each E/N/M band; thus, the bilateral average was calculated and is presented in the last column from the left. In addition, Table 2 shows the results of SA_CCO_ in the 3 E/N/M bands using the data presentation similar to Table 1. It is clear that the myogenic band was the only one with equivalent left and right prefrontal SA_CCO_. Overall, 4 bilaterally equivalent SA values were found as ISO features to characterize prefrontal ISO of the resting human brain.

**Table 1:** Grand averages of SA_HbO_ over all measurements (*n* = 130) on the left and right forehead across 3 ISO frequency bands.

Frequency band	SA_HbO, left_ (mean ± SD)	SA_HbO, right_ (mean ± SD)	Left versus right *t*-test (*P*-value)	Left versus right TOST (*P*_TOST_)	Bilateral average SA_HbO_ (mean ± SD)
Endogenic	0.16 ± 0.08	0.15 ± 0.07	0.52	< 0.01; bilaterally equivalent	**0.16 ± 0.07**
Neurogenic	0.09 ± 0.04	0.09 ± 0.04	0.76	< 0.01; bilaterally equivalent	**0.09 ± 0.04**
Myogenic	0.05 ± 0.02	0.05 ± 0.02	0.99	< 0.01; bilaterally equivalent	**0.05 ± 0.02**

Note: *P*-values and *P*_TOST_ were obtained using paired *t*-tests and TOST, respectively. See Section B in the Supplementary Material for details on TOST. The bold font marks potential features to characterize the prefrontal ISO of the resting human brain.

**Table 2:** Grand averages of SA_CCO_ over all measurements (*n* = 130) on the left and right forehead across three ISO bands.

Frequency band	SA_CCO, left_ (mean ± SD)	SA_CCO, right_ (mean ± SD)	Left versus right *t*-test (*P*-value)	Left versus right TOST (*P*_TOST_)	Bilateral average SA_CCO_ (mean ± SD)
Endogenic	0.013 ± 0.005	0.011 ± 0.005	<0.02	0.11	Left > right
Neurogenic	0.010 ± 0.004	0.009 ± 0.003	<0.04	0.08	Left > right
Myogenic	0.007 ± 0.002	0.007 ± 0.002	0.72	<0.001; bilaterally equivalent	**0.007 ± 0.002**

Note: *P*-values and *P*_TOST_ were obtained using paired *t*-tests and TOST, respectively. See Section B in the Supplementary Material for details on TOST. The bold font marks potential features to characterize the prefrontal ISO of the resting human brain.

### ISO coherence of prefrontal Δ[HbO] and Δ[CCO] in the resting human brain

**Fig. 5:**
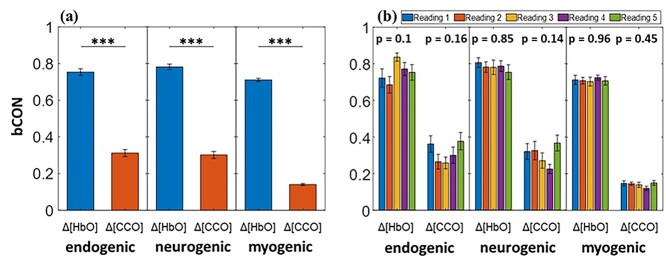
Resting-state prefrontal bCON_HbO_ and bCON_CCO_ averaged over (a) the combined set of measurements (*n* = 130) and (b) each separate set of 5 measurements (*n* = 26 per set) over E (0.005–0.02 Hz), N (0.02–0.04 Hz), and M (0.04–0.2 Hz) bands. *P*-values shown on top of each group of the bars in (b) represent one-way ANOVA results. All error bars indicate the standard error of the mean. ^*^^*^^*^P < 0.001.

Figure 5a shows the comparisons between bilateral cerebral hemodynamic connectivity and bilateral metabolic connectivity over the three ISO bands. Paired *t*-tests confirmed that bCON_HbO_ was significantly stronger than bCON_CCO_ in all the E/N/M bands. To evaluate the significant differences in these values, both bCON_HbO_ and bCON_CCO_ for bilateral connectivity were calculated for each set of 5 measurements and are plotted separately in Fig. 5b. After performing a one-way ANOVA on these 5 datasets, we confirmed that no statistically significant difference in bilateral connectivity existed among the 5 datasets at all 3 frequency bands for both Δ[HbO] and Δ[CCO], as evidenced by the *P*-values in Fig. 5b.

Specifically, the bCON_HbO_ and bCON_CCO_ values averaged over the grand group (*n* = 130) for all 3 E/N/M bands are listed in Table 3, with *P*-values obtained from one-way ANOVA (the fifth column for the left) and Tukey’s post hoc test (the sixth column). A significant difference in bCON_HbO_ (or bCON_CCO_) was observed among the 3 frequency bands. Next, we identified that bCON_HbO_ values at the E and N bands were not significantly different using Tukey’s post hoc test and were statistically equivalent based on TOST. Therefore, these 2 indices were pooled to achieve an averaged connectivity value. The same statistical analysis and spectral average over the E and N bands were achieved for the bCON_CCO_ values too, as listed in the rightmost column of Table 3. In this case, we found 2 more ISO features (i.e. bilateral hemodynamic and metabolic connectivity) that may be characteristic in the resting-state prefrontal human cortices.

**Table 3:** Resting-state prefrontal connectivity (bCON_HbO_ and bCON_CCO_) averaged over the grand data set (*n* = 130) at E/N/M band.

Bilateral connectivity	Endogenic (mean ± SD)	Neurogenic (mean ± SD)	Myogenic (mean ± SD)	ANOVA over 3 bands (*P*-value)	E versus N Tukey’s (*P*-value)	E versus N TOST (*P*_TOST_)	E, N average (mean ± SD)
bCON_HbO_	0.75 ± 0.20	0.78 ± 0.16	0.71 ± 0.10	<0.003	0.35	<0.001; laterally equivalent	**0.77 ± 0.17**
bCON_CCO_	0.31 ± 0.21	0.30 ± 0.21	0.14 ± 0.06	<0.001	0.89	<0.04; laterally equivalent	**0.30 ± 0.20**

Note: *P*-values in the fifth column from the left were obtained from one-way ANOVA to compare those at the E/N/M bands. *P*-values in the sixth column from the left were obtained from Tukey’s post hoc test to compare bCON values averaged over the E and N bands. The *P*_TOST_ values were obtained from TOST. The bold font marks potential features to characterize the prefrontal ISO of the resting human brain.

**Fig. 6:**
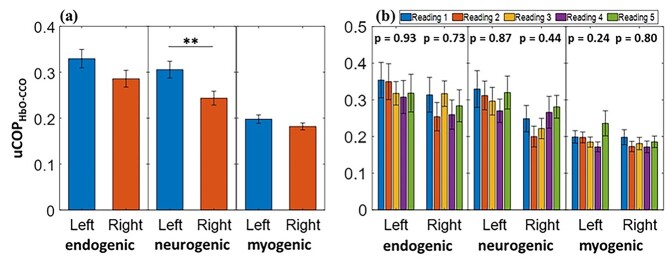
Left and right resting-state prefrontal uCOP_HbO-CCO_ obtained from (a) combined grand group (*n* = 130) and (b) separate groups (*n* = 26 each) over endogenic (0.005–0.02 Hz), neurogenic (0.02–0.04 Hz), and myogenic (0.04–0.2 Hz) frequency bands. *P*-values above each group of bars in (b) represent results from the ANOVA test. The error bars indicate the standard error of the mean. ^*^^*^P < 0.01.

Another set of coherence analyses was performed to determine the cerebral hemodynamic–metabolic coupling on each prefrontal side. Unilateral coupling between Δ[HbO] and Δ[CCO] indicates the level at which hemodynamic and metabolic ISOs are synchronized and coupled. Figure 6a shows the uCOP_HbO-CCO_ values derived from the right and left channels in each E/N/M band. Paired *t*-tests revealed that the uCOP_HbO-CCO_ values between the left and right prefrontal cortices were statistically identical in the E and M bands. Figure 6b illustrates the unilateral coupling averaged over each measurement group for the 5 repeated measurements. A one-way ANOVA over the 5 readings showed no significant difference for each coupling pair on each lateral side, as evidenced by the *P*-values given at the top of Fig. 6b. In the neurogenic band, the uCOP_HbO-CCO_ value in the left prefrontal region was significantly higher than that on the right side, indicating an intrinsic lateral difference in neurogenic oscillation in resting-state hemodynamic–metabolic coupling.

Table 4 lists the uCOP_HbO-CCO_ values over the left and right prefrontal cortices across the 3 ISO frequency bands averaged over the grand set of experiments (*n* = 130). *P*-values obtained from the paired *t*-test for uCOP_HbO-CCO, left_ versus uCOP_HbO-CCO, right_ are reported in the fourth column from the left of Table 4. In the case of no significant difference in uCOP_HbO-CCO_ between the left and right channels in the endogenic and myogenic bands, TOST for equivalence tests was perfomed with the *P*_TOST_ values reported in Table 4. Accordingly, the bilateral average of uCOP_HbO-CCO_ was calculated and represented in the rightmost column. These bilaterally averaged uCOP_HbO-CCO_ values at the E and M bands are the seventh and eighth features that we identified in this study as potential biomarkers for characterizing brain disorders in the future.

**Table 4:** Prefrontal uCOP_HbO-CCO_ values on the left and right cortical regions averaged over the grand set of measurements (*n* = 130) at each of the E/N/M bands.

Frequency band	uCOP_HbO-CCO,left_ (mean ± SD)	uCOP_HbO-CCO,right_ (mean ± SD)	Left versus right *t*-test (*P*-value)	Left versus right TOST (*P*_TOST_)	Bilateral average (mean ± SD)
Endogenic	0.33 ± 0.22	0.29 ± 0.20	0.11	0.02; laterally equivalent	**0.31 ± 0.21**
Neurogenic	0.31 ± 0.20	0.24 ± 0.17	<0.01	0.7	Left > right
Myogenic	0.20 ± 0.10	0.18 ± 0.08	0.18	0.04; laterally equivalent	**0.19 ± 0.09**

Note: *P*-values and *P*_TOST_ were obtained from paired *t*-tests and TOST, respectively, between the left and right uCOP_HbO-CCO_.The bold font marks potential features to characterize the prefrontal ISO of the resting human brain.

## Discussion

NIRS-based methods have been demonstrated and reported as well-known, noninvasive approaches to monitor the metabolic and hemodynamic activity of the human brain and thus having great potential for clinical applications (Bale et al. 2014; Buckley et al. 2014; Chen et al. 2020). Because NIRS quantifies only changes in cerebral hemodynamics, it is not applicable in clinical practice for disease diagnosis or monitoring when the human brain is in a resting state. For instance, diffuse correlation spectroscopy and functional NIRS only detect the relative blood flow index and relative changes in hemoglobin concentration, respectively (Durduran and Yodh 2014).

To address this weakness of NIRS, we developed a frequency domain analysis to determine the SAs and coherence indices for each ISO time series on both sides of the prefrontal cortex. While both the Δ[HbO] and Δ[CCO] time series in the resting brain expressed changes with respect to a baseline point, each SA of the oscillation would be an absolute value, in μM, and signified the respective oscillation amplitude. Because the analysis was performed in the frequency domain, SA values in the E/N/M bands denoted oscillation magnitudes of HbO and CCO at the 3 respective rhythms. In addition, coherence indices are absolute values in the range of 0–1, regardless of the unit. It represents the degree of oscillatory similarity between the 2 neurophysiological rhythms. Accordingly, we developed and demonstrated a low-cost, portable, 2-channel bbNIRS system to record cerebral hemodynamic and metabolic ISO activity over the prefrontal cortex of healthy young humans with a relatively large sample size (*n* = 26). The recorded signals were analyzed using a frequency domain approach to quantify the SA and connectivity/coupling of ISO in the resting human brain. As discussed below, this study enabled us to prove and support our hypothesis by achieving absolute quantification of ISO-resolved hemodynamics and metabolism in the resting-state prefrontal human cortices. The quantified metrics were shown to be relatively stable and thus may have great potential to be developed as biomarkers for the characterization, diagnosis, and monitoring of certain brain disorders.

### ISO SAs of prefrontal Δ[HbO] and Δ[CCO] as brain-state features

The ISO (0.005–0.2 Hz) consists of 3 distinct frequency components: endogenic (0.005–0.02 Hz), neurogenic (0.02–0.04 Hz), and myogenic (0.04–0.2 Hz). Each frequency band is associated with a specific neurophysiological activity in the healthy human brain (Kvernmo et al. 1999; Zhang et al. 2002; Newman et al. 2009; He et al. 2018). Thus, abnormal brain activity and neurological disorders in the human brain are associated with impaired or irregular patterns of cerebral hemodynamic and metabolic ISOs. Several studies have reported a relationship between ISO impairment and cardiovascular disease, Alzheimer’s disease, hypertension, and stroke (Girouard and Iadecola 2006; Deanfield et al. 2007; Marco et al. 2015).

In this study, we quantified ISO-resolved SAs of prefrontal oxygenated hemoglobin (SA_HbO_) and oxidized cytochrome *c* oxidase (SA_CCO_) in healthy young humans. Next, we demonstrated that the SA_HbO_ and SA_CCO_ values averaged over 26 participants with 5 different repeated measurements were relatively stable and consistent, as evidenced by the ANOVA results shown in Fig. 4b. Furthermore, as shown in Fig. 4a and Table 1, the average SA_HbO_ values over the 2 lateral sides of the prefrontal cortex were statistically equivalent, indicating similar levels of hemodynamic oscillation in the bilateral prefrontal cortices in all vasomotion-derived ISO (i.e. E/N/M) bands. Thus, a set of standard prefrontal SA_HbO_ values for each of the 3 ISO bands can be established and further examined as features that characterize abnormal brain functions. As shown in Fig. 4c and Table 2, identical or equivalent levels of grand-averaged SA_CCO_ between the 2 prefrontal cortices in the myogenic band are unambiguous, implying that this metric can be considered another potential prefrontal feature.

It is worth noting that the grand-averaged SA_CCO_ indices were significantly larger on the left than on the right prefrontal side in both the E and N bands. This observation may imply higher metabolic activity in endogenic and neurogenic rhythms on the left side than on the right side of the resting prefrontal cortex. As reported by Liu et al. (2009), the dominant source of resting-state lateralization in human brain activity is the default mode network (DMN), which is associated with internal thoughts. The study (Liu et al. 2009) provided evidence that the DMN is predominantly active in the left prefrontal cortex in the resting state, especially in right-handed participants. The implication of our observation matched well with the results of Liu et al. (2009) because most of our participants were right-handed and would give rise to higher anterior default-mode activity in the left prefrontal cortex (Liu et al. 2009; Nielsen et al. 2013). Furthermore, as illustrated in Fig. 4a and c, the endogenic band had higher values for SA_HbO_ and SA_CCO_ than those of the other 2 bands. This phenomenon has also been reported in other studies that have used different NIRS systems and analysis algorithms as well (Obrig et al. 2000; Rivadulla et al. 2011; Salvi et al. 2018).

### Cerebral hemodynamic and metabolic ISO connectivity/coupling as features

As shown in Fig. 5a and Table 3, robust bilateral connectivity of prefrontal hemodynamics (bCON_HbO_) was identified over the E/N frequency bands in 26 young healthy human subjects at rest, over 5 repeated measurements. This high level or index of connectivity may imply synchronized hemodynamic activity mediated by endothelial cells and inter-neurons on lateral prefrontal regions (Elwell et al. 1999; Razavi et al. 2008; Bosch et al. 2017). In contrast, bCON_CCO_ showed a significantly lower level of bilateral functional connectivity than bCON_HbO_. A lower level of bCON_CCO_ can be expected because unilateral CCO activity is locally driven by oxygen consumption and/or mitochondrial metabolism within neurons, has specific functions distinct from those of the other lateral prefrontal cortex, and has less need to link to the other side. Similar to bCON_HbO_, bCON_CCO_ was statistically identical over the E and N frequency bands. These observations are in good agreement with those of a recent study by our group, which had a smaller sample size and utilized a different 2-bbNIRS setup and analysis methods (Wang et al. 2022). Furthermore, Fig. 5b illustrates the ANOVA-driven results with nonsignificant differences among the bCON_HbO_ (or bCON_CCO_) values over 130 measurements. These high stabilities suggest and support the possibility of using these metrics as new neurophysiological features to characterize the human brain state.

As the final metric, the unilateral hemodynamic–metabolic coupling (uCOP_HbO-CCO_) in the right and left channels is plotted in Fig. 6a. As shown in this figure and reported in Table 4, no significant difference in uCOP_HbO-CCO_ between the 2 lateral sides existed at both the E and M bands; thus, bilaterally pooled coupling indices at both E/M bands could be achieved. Similar to the other metrics shown above, Fig. 6b illustrates the ANOVA-driven results of the statistically nonsignificant uCOP_HbO-CCO_ values/indices bilaterally over 130 measurements in the E and M bands. Thus, prefrontal uCOP_HbO-CCO_ in the E and M bands can be included and tested as resting-state features in future clinical applications.

In contrast, the neurogenic component of uCOP_HbO-CCO_ was significantly higher in the left prefrontal cortex than in the right prefrontal cortex. This observation may imply a higher vascular–metabolic interactivity on the left prefrontal cortex than on its contralateral side. Given that most of our participants were right-handed, higher hemodynamic–metabolic coupling in the left prefrontal cortex would be expected (Liu et al. 2009; Nielsen et al. 2013).

As mentioned in the Introduction section, relaxation–contraction cycles of blood vessel walls are expected to be the driving force for the ISO rhythms of cerebral hemodynamics. However, the driving force of the ISO for CCO is unclear. A recent study using fMRI and PET demonstrated a strong correlation between slow oscillations (0.01–0.1 Hz) of hemodynamics and metabolism in the brain (Deng et al. 2022). Specifically, the authors concluded that metabolic demand for glucose and oxygen regulates low-frequency hemodynamic fluctuations. Because of the strong correlation and thus close coupling between HbO and CCO constituents, we speculated that the 3 E/N/M oscillations originating from slow vasomotion may be passed or translated to mitochondrial (CCO) oscillations at the E/N/M rhythms.

### Eight measurable features of prefrontal ISO

In the Results section, we confirmed our hypothesis that prefrontal cortical connectivity and coupling of the ISO can be quantified using 2-bbNIRS as features that reflect the brain state. Specifically, through the aforementioned content, we demonstrated several stable or consistent metrics based on prefrontal bilateral connectivity and unilateral coupling of ISO. Based on the analyses and discussions presented above in the Discussion section, we list 8 metrics as measurable features in Table 5. These features can be further studied and validated using a larger sample size of both healthy human participants and patients with certain brain disorders.

**Table 5:** Measurable ISO features for characterization of the prefrontal human brain at rest.

ISO features (frequency band)	Average over two lateral sides (μM)
SA_HbO_ (E)	0.16 ± 0.07
SA_HbO_ (N)	0.09 ± 0.04
SA_HbO_ (M)	0.05 ± 0.02
SA_CCO_ (M)	0.007 ± 0.002
ISO features (frequency band)	Connectivity between two lateral sides
bCON_HbO_ (E/N)	0.77 ± 0.17
bCON_CCO_ (E/N)	0.30 ± 0.20
ISO features (frequency band)	Average over two lateral sides
uCOP_HbO-CCO_ (E)	0.31 ± 0.21
uCOP_HbO-CCO_ (M)	0.19 ± 0.09

### Limitations

First, the relatively low sampling frequency and short data collection duration (i.e. 7-min) prevented us from achieving high-frequency resolution, which may have led to low accuracy in SA and coherence calculations in the low-frequency range, especially in the endogenic band. It is suggested to have a longer measurement duration, for example, 10 min or longer. Second, our bbNIRS system was sensitive to motion; the eyes-closed resting-state protocol may have caused sleepiness in the participants during the measurements. Finally, our quantified results or metrics may be contaminated by the extracranial layers of the human head. It is known that fNIRS signals obtained over the scalp of human participants are contaminated by extracranial layers, namely, the human scalp and skull. To minimize this potential confounding factor, additional optical channels of fNIRS with a short source-detector (S-D) separation (commonly ~0.8 to 1.2 cm) have been used for systemic noise removal in task-evoked hemodynamic studies (Zhang et al. 2007; Tian et al. 2011; Yucel et al. 2015; Zhou et al. 2020; Noah et al. 2021), where a cortical region was activated by stimulating tasks. However, most fNIRS-based studies for quantifying resting-state functional connectivity (RSFC) have not developed an appropriate methodology to remove this confounding effect (Cai et al. 2018; Hu et al. 2021). It is reported only recently that RSFC can be quantified more accurately with a short S-D reading correction than without correction (Paranawithana et al. 2022).

### Future work

In future work, to enable a longer-period and less-artifact recording from the human brain, improvements are needed in the bbNIRS setup, measurement protocol, and computational methods to reduce movement artifacts and systemic/physiological noises. In addition, it is necessary to consider the implementation of short-distance channels in bbNIRS to remove the possible contamination of extracranial layers from the determined results.

The current study included only healthy controls without any disease-related patients; thus, it was an exploratory study (Hallingberg et al. 2018). While we believe that the identified ISO features are good neurological representations of the human brain, proof-of-principle or confirmatory research must be conducted for these features to become biomarkers of neurological diseases. Such studies include 2 parts. First, the features need to be stable, reliable, and with known or tested dependence on age, sex, and brain state. All of these quantifications need to be obtained using a statistically large sample size of healthy controls. Second, the features must be efficient in significantly classifying controls and patients with selected neurological disorders.

## Conclusion

In this study, we hypothesized that 2-bbNIRS, along with frequency domain analysis, enables the quantification of prefrontal cortical connectivity and coupling of ISO in the resting human brain. To test this hypothesis, we implemented 2-channel bbNIRS and performed bilateral, prefrontal, 7-min measurements in an eyes-closed resting state in vivo from 26 young and healthy participants, repeated 5 times over 5 weeks. The measured time series were analyzed using a frequency-domain approach to detect cerebral hemodynamic and metabolic ISO in 3 endogenic, neurogenic, and myogenic frequency bands at rest. Specifically, coherence analysis facilitated the quantification of bilateral connectivity and unilateral hemodynamic–metabolic coupling in the human prefrontal regions. Accordingly, we identified eight stable resting-state ISO-specific metrics or features, including bilaterally averaged SA_HbO_ in all 3 bands, bilaterally averaged SA_CCO_ in the M band only, and bilaterally connected network metrics for both bCON_HbO_ and bCON_CCO_, each of which was statistically identical in the E and N frequency bands, respectively. The last 2 features were the bilaterally averaged coupling indices of uCOP_HbO-CCO_ in the E and M bands, given that the coupling indices were statistically equivalent for both lateral sides. All 8 metrics as features showed a statistically stable level for 130 measurements. In short, this exploratory study developed a quick, low-cost, and effective methodology for exploring several prefrontal cortical connectivity and coupling features in the resting, healthy, and young human brains. The framework reported in this paper has demonstrated the potential of ISO features to be translatable for future clinical applications, while further confirmatory studies are needed before these features become effective biomarkers to identify certain neurological disorders.

## Supplementary Material

Supplementary_material_for_Resting_state_CCC_July_16_2022_final_tgac033

## Data access statement

The research data supporting this publication are available upon request from the corresponding author.
