# Supplementary material for: Prefrontal cortical connectivity and coupling of infraslow oscillation in the resting human brain: a 2-channel broadband NIRS study
Source: Cereb Cortex Commun. 2022 Aug 4;3(3):tgac033. doi: 10.1093/texcom/tgac033 (PMC9441674; doi:10.1093/texcom/tgac033)
Supplement: Supplementary_material_for_Resting_state_CCC_July_16_2022_final_tgac033 [file supplementary_material_for_resting_state_ccc_july_16_2022_final_tgac033.docx]

Prefrontal Cortical Connectivity and Coupling of Infraslow Oscillation in the Resting Human Brain: *A Two-Channel Broadband NIRS Study*

Sadra Shahdadian, Xinlong Wang, Shu Kang, Caroline Carter, Akhil Chaudhari,

and Hanli Liu^*^

**Supplementary Material**

**A. Steps for Frequency-Domain Data Analysis of Prefrontal Δ[HbO] and Δ[CCO] at Rest**

Figure S1 shows a detailed flow chart and demonstrates the frequency analysis steps for a pair of signals, namely, Δ[HbO] and Δ[CCO]. These steps consist of two major functions available in the FieldTrip toolbox (Popov, Oostenveld, and Schoffelen 2018; Oostenveld et al. 2011), namely, “ft_freqanalysis” for amplitude and phase quantifications of each signal, and “ft_connectivityanalysis” for coherence quantification between the two signals.

As illustrated in this figure, the input for function “ft_freqanalysis” can be a single time series of two signals, followed by frequency-domain analysis using the multi-taper method (mtm) with *k* tapers. In this step, each tapered time series was subjected to a fast Fourier transform (FFT) to obtain the first set of outputs for this function. A total of k tapers resulted in k sets of complex numbers with their respective amplitudes and phases in the frequency range of the signal. Next, the mtm-based power spectral density (mtm-PSD) and spectral power were obtained for the input signal by averaging k sets of spectral powers. Accordingly, the respective spectral amplitude (SA) was calculated by taking the square root of the mtm-derived spectral power of the input time series (i.e., Δ[HbO] or Δ[CCO]) in the frequency band of interest. These steps are outlined by blue and orange boxes in Figure S1 for Δ[HbO] and Δ[CCO], respectively.

In addition, the outputs of *k* complex members from “ft_freqanalysis” are used as the inputs for function “ft_connectivityanalysis.” This function quantifies the coherence index between the *i*th tapered output of the first signal and the corresponding (i.e., the *i*th) tapered output of the second signal. Each spectral coherence between a pair of temporal signals with the same *k*th taper was averaged over all *k* tapers, leading to the quantification of bilateral connectivity (i.e., bCON_HbO_ and bCON_CCO_) and unilateral coupling (i.e., uCOP_HbO-CCO_) in all three E/N/M frequency bands, as marked by the three green boxes in the figure.

**
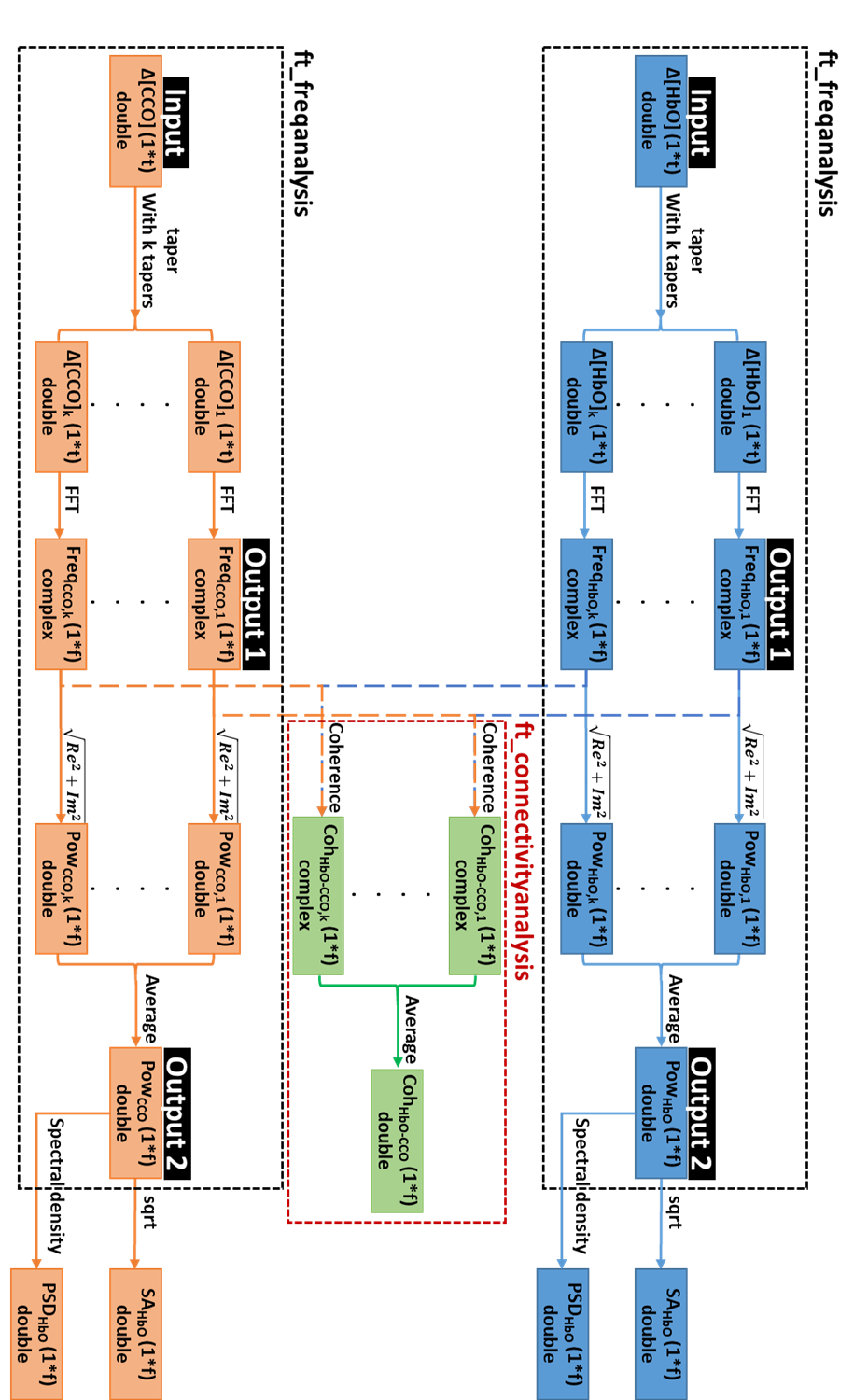
**

**Figure S1 Schematic flow chart of spectral analysis for the quantification of SA and coherence.** For demonstration, two time series, Δ[HbO] and Δ[CCO], are used as separate input signals with a time period of ‘t’. Blue and orange blocks represent frequency analysis steps operated on signal 1 (i.e., Δ[HbO]) and signal 2 (i.e., Δ[CCO]), using “ft_freqanalysis” function (outlined by black dashed boxes). The word of “double” and “complex” indicates a real number with double precision and a complex number, respectively. Furthermore, green blocks represent connectivity analysis steps operated on the frequency-domain outputs of the two signals, using “ft_connectivityanalysis” function (red dashed box).

**B. Statistical Analysis for the Test of Equivalence Using the Two One-Sided Tests (TOST)**

The two one-sided tests (TOST) analysis was utilized to evaluate the equivalence of the features that did not show a significant difference between right and left (for SA_HbO_, SA_CCO_, and uCOP_HbO-CCO_) or between endogenic and neurogenic (for bCON_HbO_ and bCON_CCO_).

In each set of tests, the null hypothesis was that the means of the two samples were not equivalent. In principle, TOST returns two p-values (one for each side of the test). Thus, if both p-values obtained from this test are lower than the significance level threshold (i.e., 0.05), we can reject the null hypothesis and conclude that the means of the two samples are equivalent. In this method, a pair of lower and upper bounds for the feature must be determined, which is closely related to the hypothesis or the predefined effect size. In our study, for each feature in each frequency band, we defined the upper and lower bounds for the equivalence test as *M*×(1 ± 0.2), where *M* is the average value of the two means of the two samples. In the case of statistically significant equivalence between the two samples (i.e., p*_TOST_* < 0.05), the average of the tested features was also reported. A function of “TOST” in MATLAB was used in our analysis for TOST in this step of analysis (Anisha Retrieved July 15, 2022. ).

**C. Decomposition of a [HbO] Time Series into Three ISO Frequency Bands**

Figure S2(a) shows an example of the Δ[HbO] time series from one channel of 2-bbNIRS in the dataset of a subject. Figures S2(b) to S2(d) were obtained after applying a Butterworth band-pass filter to the trace in Figure S2(a) using separate bandwidths of three endogenic (E:0.005-0.02 Hz), neurogenic (N:0.02-0.04 Hz), and myogenic (M:0.04-0.2 Hz) frequency ranges. This set of figures illustrates how different ISO components contribute to the composition of the wideband (0.005–0.2 Hz) original signal, as shown in Figure S2(a).


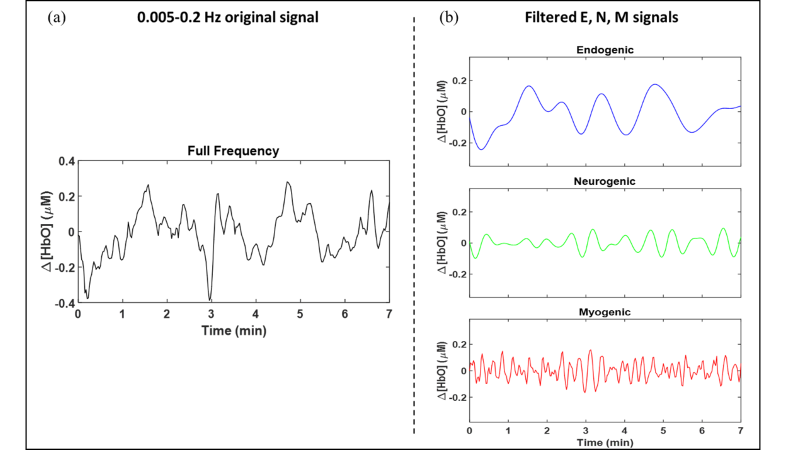


**Figure S2** (a) A 7-min time series of Δ[HbO] derived from one channel of 2-bbNIRS of a subject’s dataset. The three panels on the right were obtained after Butterworth band-pass filtering of the original signal in the three predefined E/N/M bands, namely, 0.005-0.02 Hz, 0.02-0.04 Hz, and 0.04-0.2 Hz, respectively.

**References:**

Anisha. Retrieved July 15, 2022. . "TOST(sample1, sample2, d1, d2, alpha)." In. <https://www.mathworks.com/matlabcentral/fileexchange/63204-tost-sample1-sample2-d1-d2-alpha> , MATLAB Central File Exchange. .

Oostenveld, Robert, Pascal Fries, Eric Maris, and Jan-Mathijs Schoffelen. 2011. 'FieldTrip: open source software for advanced analysis of MEG, EEG, and invasive electrophysiological data', *Computational intelligence and neuroscience*, 2011.

Popov, T., R. Oostenveld, and J. M. Schoffelen. 2018. 'FieldTrip Made Easy: An Analysis Protocol for Group Analysis of the Auditory Steady State Brain Response in Time, Frequency, and Space', *Front Neurosci*, 12: 711.
